# A National Pension Fund for Hospital Officials and Nurses

**Published:** 1887-03-12

**Authors:** 


### A National Pension Fund for Hospital Officials and Nurses.

The Views of Secretaries, Sisters, Nurses, and others.

By a Practical and Experienced Secretary.

I have long felt that a great want has not been supplied in connection with our hospital system, in the shape of a scheme of a provident nature, by which our hospital officials, nurses, and servants could make a provision during sickness, and possible loss of income, and the inevitable period of age, when the income must cease from the inability of the individual to command a salary or wage. The establishment of The Hospital has, I venture to think, opened a medium for the ventilation of an idea, and the possible consummation of a hope, of one for many years connected with hospital work. I respectfully ask that you will open your valuable columns for the discussion of the proposals I now lay before your readers. We all know that the salaries and wages of our hospital officials and servants are not of such an elastic nature that they will stretch to the extent of providing an adequate sum to produce an income for old age ; nor is there a great margin for any large provision for the family, in the event of the decease of the bread-winner. I therefore feel that where the interests of so large a body of people are concerned—practically servants of the public welfare—that the time has come when a comprehensive scheme could be formulated whereby for a small weekly, monthly, or annual outlay the various accidents and eventualities of the every-day life of the toilers in our hospitals could be provided against.

My idea is, that a provident system could be established on a purely commercial ground, by which hospital officials and servants could secure pensions for old age, allowances during sickness, weekly payments whilst recovering from accidents, insure their lives in the ordinary way, and against death by accident. A separate company might be floated to cover all these heads, or a high-class established office influenced to open a separate branch, by the issue of additional stock, to meet the necessary outlay for salaries, rent, etc. I believe that the large interests involved in our hospital and infirmary machinery would provide a constituency sufficient to float a company on either of the above bases, and I beg leave to lay this question before your readers, with the sincere hope that something may be done ; but if my suggestion falls to the ground, I shall have had the satisfaction of having tried to help my fellow-workers.

Newton H. Nixon.

University College Hospital.

---

By a Sister.

I have been much interested in the letters on "The National Pension Fund" in last week's Hospital, and think every nurse should be grateful for your efforts on behalf of the scheme. I feel sure a great number of nurses would be most thankful if a really practical scheme for giving them a pension when unable to work, through age or confirmed illness, were established. But I believe few know that such a scheme has been even proposed. I think it would be found that many do care about the subject, though unable to move in the matter themselves, and believe that nurses would remain longer in the work were they sure of a pension when no longer fit for its arduous duties. Mr. R. G. Salmond states there are 10,000 nurses in the United Kingdom, and suggests they each give 1*s*. a year to the "Trained Nurses' Annuity Fund." I think they might be induced to subscribe or collect from 10*s*. to £2 each during this Jubilee year, to help found a "National Pension Fund," and to continue the subscription yearly, were it made a "Provident" fund, as Mr. Burdett suggests.

A Hospital "Sister."

---

By a Trained Nurse.

You say you would like to hear the view nurses take about the Pension Fund, or I should not have written so soon. I am very much afraid the majority have never thought anything about it at all. It is also quite true a large number marry, or are obliged to give up nursing through bad health. But it is only too well known that many make little provision for a long sickness or old age. And I believe it is not always their fault. A nurse ought not to do without a holiday every year ; if she does, she nearly always breaks down. Three weeks or a month make a great hole in a year's wages ; for I believe most nurses will agree with me that they have either to keep themselves or make presents to their friends. Again, if a nurse is taking a good salary, she feels bound to give help to relations who are not so well off as herself—all honour to her, —and she thinks each year, when the next comes she will save more ; but as time goes on her account at the bank does not increase much, and when sickness comes a long rest is needed, which obliges her often to draw on this. I am not saying anything of nurses who live above their means, spending their money on extravagant clothes, over-feeding, and—dare I say ?—drinking. These I must leave to those who are in a higher position than myself, who often set them the example. If a pension fund were started, and nurses could have what money they had paid in returned to them in case of marriage or changing their calling, I think many would be glad to subscribe, if the Superintendents of hospitals and institutions brought it to the notice of those under them.

B. S.

---

By One of the Public.

In further looking into the rules and regulations of the "Trained Nurses' Annuity Fund," I note that "on election each annuitant shall pay the sum of £15 towards the General Fund," and that those who have saved that sum will have a "preference" over other candidates. I certainly think such rules as I referred to last week, and this one, help to exclude the majority of the nurses from its benefits, and limit the objects of the Fund considerably. Many nurses break down long before attaining the age of fifty, and before the qualifying term of fifteen years' service. A large proportion are well-born and educated gentlewomen, who, through family bereavement or other causes, have chosen this excellent and noble vocation to assist in supporting dependent relatives, and thus are absolutely unable to save the £15 upon which their eligibility to apply for a grant from the existing Annuity Fund is based. If Mr. Salmond's Society is unable to extend the benefits of his fund, so as to include all trained nurses, who, through the strain on the constitution consequent upon incessant watching, vitiated air, etc., have become incapacitated from further service, cannot we this year start a fund for benefiting these noble woman who, at the greatest risk to themselves, give up the best years of their lives to so carefully nursing their sick brothers and sisters? The medical profession, I feel certain, would generously second any efforts made in this direction, the nurses, when possible, giving what they could afford, and our wealthy neighbours being asked to give a small annual subscription. I should be pleased to receive any suggestion with this object in view, and thanking you for your valuable help in the cause. W. SNAPE.

109, Sussex Road, Holloway, N.

